# Profiles of and practices in crisis resolution and home treatment teams in Norway: a longitudinal survey study

**DOI:** 10.1186/1752-4458-5-19

**Published:** 2011-08-30

**Authors:** Bengt Karlsson, Marit Borg, Marthe Eklund, Hesook Suzie Kim

**Affiliations:** 1 Buskerud University College, Department of Health, Box 235, 3601 Kongsberg, Norway

## Abstract

**Background:**

Crisis resolution and home treatment (CRHT) is one of the more recent modes of delivering acute mental health care in the community. The objective of the study was to describe the standardizations and variations in the CRHT teams in Norway in order to gain knowledge regarding the structures and processes of CRHT teams.

**Methods:**

A longitudinal survey of five CRHT teams in Norway was carried out for a period of 18 months with two sets of questionnaires-one for CRHT team profiles for a bi-yearly survey and the other for services and practices of CRHT teams for a monthly survey.

**Results:**

The five CRHT teams were configured by a set of common basic characteristics in their operations, while at the same time were variant in several areas of the teams' structures and processes. Significant differences among the teams were evident in terms of the structural aspects such as service locality, staffing and team make-up, caseload, service hours, and travel time, and the process aspects such as the number of referrals received, referral source, admission, service duration, and discharge destination. These variations are reflected upon the perspectives regarding the nature of mental health crisis, the conflicting policies in mental health services, and the nature of home-based mental health care.

**Conclusions:**

The diversity in the way CRHT teams are established and operate needs to be examined further in order to understand the reasons for such variations and their impact on the quality of services to service users and in relation to the total mental health service system in a community.

## Background

Comprehensive changes have been seen in the mental health service systems during the last decades with the intention to benefit service users and their families. A major challenge in these developments has been to establish accessible and competent acute mental health services in lieu of or in combination with acute in-patient care. One intervention has been crisis resolution home treatment team (CRHT), one of the so-called 'functional teams' developed in United Kingdom as part of The National Service Framework (NSF) [1,2]. In line with World Health Organization's and the European policies' emphasis on community based treatment and rehabilitation, the overall objective of these teams is to offer comprehensive treatment and support in people's home environment and prevent hospital admission. CRHT teams attempt to provide an alternative to hospital admission, robust psychosocial as well as psychiatric assessments, gate-keeping of admissions, and opportunities to resolve crisis in the contexts of their occurrence [1-4].

In Norway CRHT was introduced as part of the National Action Plan for Mental Health [5,6]. Although the concept of CRHT in the Norwegian context aligns with the ideas embedded in the above statement, it differs from the way CRHT is specified in terms of the modes of operation, staffing and team provision by the UK standards [1,7,8]. It is apparent that the term CRHT connotes different meanings in various national and local contexts [1,4,7,9]. CRHT is the term used in this paper as the subjects of this study were the community-based mental health teams established as CRHT teams in the regional centers in Norway [9]. Surveys in England [1,2,4,7] and Norway [9] reveal a great divergence in team structure, clients seen, outreach practices, operational hours, caseloads, and 'gate keeping' models in CRHT teams. Team profiles and standards of practice vary a great deal, and the term 'crisis resolution and home treatment team' conveys a multifaceted and unclear picture. A literature-review by Sjølie et al. [8] revealed that most of the published articles on CRHT focus on structural issues pertaining to the development of home treatment services and on macro-level outcomes such as cost-effectiveness and admission rates, which have political, economic, and practical implications. However, there is a paucity of research regarding the structures and processes of CRHT teams in actual operations over time, as well as a lack of research of service-user experiences and satisfaction on key areas of service provision [1,4,10].

In Norway, a national strategy was formulated to establish crisis resolution and home treatment teams at each of the 78 community mental health centers (DPS) by the end of 2009. In order to implement the CRHT teams a set of guidelines were worked out, based on international experiences [6]. The key service characteristics of CRHT teams in Norway were defined as (a) brief responding time, (b) provision of assessment and direct care in the context of home and family, (c) working in partnerships with relevant health and social welfare providers, and (d) assessment and course of action that may include inpatient treatment, home treatment, crisis resolution by the team and next-level referrals to health and social services [6,10]. Although these criteria describe the way teams are encouraged to work and achieve their targets, the practices are less well defined and seem greatly influenced by local factors, such as allocated recourses, interdisciplinary staff, urban or rural, and the organization and priorities of the total mental health services in the area.

The aims of this study were to explore and describe the profiles and practices of five CRHT teams in a health region in Norway by 1) identifying the basic characteristics of the five teams; and 2) describing the services provided by these teams over a period of 18 months. The ultimate purpose was to gain an in-depth understanding about the nature of variations in the structures and processes of CRHT teams.

## Methods

### Design

A descriptive, quantitative longitudinal survey with two sets of questionnaires, one for team profile and the other for the services, was carried out to address the research questions. The data were collected from five CRHT teams established in the Health South Region in Norway over a period from May 2008 to June 2010. The data with the first questionnaire for the CRHT team profile were collected every six month for five times, while the data for the second questionnaire for the services and practices of the CRHT team were collected monthly for 25 consecutive months. Thus the total data set constitutes the team profile data for 5 time points and the team service data for 25 time points.

### Participants

In the spring of 2008 all of the established CRHT teams in the previous Health South Region in Norway (the designation of the Health South Region has changed somewhat in 2009 and 2010) were asked to participate in the study, with the information that participation would involve filling out the questionnaires at regular intervals by a permanent staff member/secretary. There were ten teams, of which five teams agreed to participate in the study. The teams declining to participate gave the reason either that they were not able to spend the time required for the paper work or that their teams were not settled and ready to participate in such a study.

### Instruments

Two questionnaires were developed in consultation with Professor Mervyn Morris of BCU, drawing on a similar study in the UK [M. Morris, personal communications, 2007-2009], adjusting them for the Norwegian context by the research team. The questionnaire for CRHT team profile (the profile questionnaire) addresses the organizational and structural characteristics of the CRHT team, including general service profiles, structure of the team, and how the team works. The questionnaire for CRHT service (the service questionnaire) addresses the team's actual services in terms of referrals and sources of referrals, user registration and user demographics, service duration, and discharge destinations for a given month. Both questionnaires asked for aggregated data rather than individual service user data.

### Data Collection Procedures

The teams consenting to participate in the study were invited to a meeting to inform and discuss the study's procedures and the responsibilities of the teams regarding the study. Each team also was asked to identify a responsible staff person for the surveys, who was telephoned by a member of the research team at each data collection point to remind the submission of the questionnaire. Additional telephone calls were made when the questionnaires were not received within two weeks of the due dates. Additional meetings with the representatives of the participating teams were held every six months during the data collection period in order to address problems related to the data collection. The monthly data from the pilot period of first seven months were not included in the final data set. Therefore, the data for this study include five bi-yearly team profile reports and 18 monthly service reports.

### Data Analysis

The data were analyzed by the statistical software PASW for Windows version 17.0. for SPSS for descriptive statistics and chi square tests.

### Ethics

The Regional Medical Research Ethics Committee, Health Region II (South) of Norway and the Norwegian Social Science Data Services on behalf of The National Inspectorate approved this study.

## Results

### The Teams-Profiles

As shown in Table 1 the total numbers of professional staff on the teams varied from 4 to 14 in the 18 months period. The majority of the professional staff was nurses. Four out of 5 teams had psychologists and 4 out of 5 teams had a psychiatrist on their staff. Although there were some fluctuations in the numbers of the professional staff at five data points, these did not vary greatly, suggesting some stability in staffing.

**Table 1:** Professional staff in the CRHT teams

Professional Staff	Team 1	Team 2	Team 3	Team 4	Team 5
Total Number of Staff	10 or 11	9-14	5-7	6-9	4
Physician	0	1	1 or 2	1	1
Psychologist	1	2-4	None or 1	0-2	0
Nurse	7	4-6	2	5 or 6	3
Social educator	1 or 2	None or 1	0	0	0
Social worker	1	None or 1	0	0	0
Health worker	0	None or 2	0	0	0
Team leader	0	0	1	0	0
Nursing assistant	0	None or 1	1	0	0

Postgraduate training among the team members was comprehensive as shown in Table 2.. The most typical training was in mental health care, while training in cognitive therapy, family and network therapy as well as psychosis and early intervention also were represented.

**Table 2:** Postgraduate training of the staff in the CRHT teams

Professional Training	Team 1	Team 2	Team 3	Team 4	Team 5
Mental health care	8 or 9	3-5	1	5 or 6	2
Psychosis & early intervention	0	None or 1	1	0-2	0
Cognitive therapy	2 or 3	0-2	0	0-3	1
Psychiatry	0	1	1	0 or 1	0 or 1
Clinical psychology	0	1	0	0	0
Family and network yherapy	None or 1	0	0	None or 1	0
Management	None or 1	0	1	None or 1	0
Group therapy	0	0	0 or 1	0	0-2

The basic characteristics of the 5 CRHT teams are shown in Table 3. Three teams have their offices in the city centres, having the responsibilities for the surrounding municipalities, while two teams are located in the rural areas having responsibilities for larger geographical areas and greater number of municipalities. Two teams were established prior to 2007, while three teams were established in 2007. The ratios of the population base to professional staff varied from over 10,000 to 1 in three teams to the low of 1,770 to 1 in Team #3. The staffing level for the teams was higher for the teams in the urban areas but the ratios of the staff to population were much lower in the rural teams, suggesting the teams servicing the urban areas with large populations even with higher numbers of professional staff would have greater service loads. This was accounted by the lower ratios of the total CRTH cases over a period of 18 months to the professional staff in the rural teams (15 to 1 in Team #3 and 28 to 1 in Team #5) compared to those in the urban teams (172:1, 515:1 and 298:1 in the three urban teams).

**Table 3:** General features of the CRHT teams

Features	Team 1	Team 2	Team 3	Team 4	Team 5
Date of team establishment	2007-01-09	2005-09-01	2003-10-03	2007-09-01	2007-05-01
Total number of communities covered	5	4	4	15	8
Urban/Rural	Urban	Urban	Rural	Urban	Rural
Population base	130,000	150,000	12,390	100,000	25,700
Total number of consultations for the 18 months	1,889	7,207	107	2,682	110
Total number of professional staff on CRHT team	10-11	9-14	5-7	6-9	4
Ratio-Population to professional staff	11,818:1	10,714:1	1,770:1	11,111:1	6,425:1
Ratio-Professional Staff to CRHT cases for the 18 months	172:1	515:1	15:1	298:1	28:1

Table 4 provides the general profiles of the five CRHT teams obtained from the five bi-yearly reports on the profile questionnaire. As shown in this table, these CRHT teams varied a great deal in terms of the ways the services were provided. The average monthly case loads for the teams varied from the low of 6 to the high of 70. Hence, the average case loads per month per professional staff would be 2 for Team #1, 5-8 for Team #2, 8-10 for Team #3, 8-12 for Team #4, and 1.5 for Team #5, suggesting two polar levels of service intensity for the teams with two teams with about 2 cases and three other teams with 5-12 cases per professional staff per month. The target response time to referrals was either 24 or 48 hours, and the average amount of time spent per episode ranged from 60 minutes for two teams to 120 minutes, 264 minutes, and 390 minutes for one team each. The average days in service per case varied among the teams, with two teams reporting 1 day as the average while three teams reporting 10 to 17, 1 to 32, and 1 to 60 as the averages. Similarly, the maximum duration of services per case also varied a great deal from the low of 35 days to the high of 180 to 365 days. This means that the maximum service could be two month to one year. The maximum distance to travel to provide services and the travel time spent on services were higher in the rural teams than in the urban teams as expected.

**Table 4:** CRHT Teams General Service Profiles

Profile items	Team 1	Team 2	Team 3	Team 4	Team 5
Average case load per team per month	20	70	54	69	6
Target response time in hours	24	48	48	24	48
Average treatment-time in minutes per patient	60	264	120	390	60
Average days in service per patient	10-17	1-32	1	1-60	1
Maximum duration of services in days	60	365	35	180	60
Maximum travel time for service in minutes	60	35	90	240	90
Maximum distance for service in Km	40	30	80	270	95

All five teams provided a service user with a named professional staff as his/her case coordinator. However, Teams #1 and #4 adopted 'teamwork approach' to manage the caseload while Team #3 did not adopt it, and Teams #2 and #5 reported to vacillate between adopting and not-adopting 'teamwork approach' during the five report points. Teams #1 and #4 consistently applied teamwork approach by having daily, weekly team meetings for diagnoses and treatment plans, regular review team meetings, and also having non-team members to attend their review meetings. Teams #3 and #5 did not have team meetings, while Team #2 reported having daily, weekly, or review team meetings sometimes.

**Figure 1:**
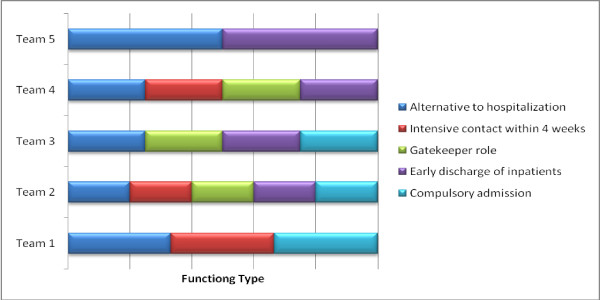
**The profiles of the CRHT team functioning**.

In regards to the teams' functioning, all five teams reported their teams to be able to provide an alternative to hospitalization to service users experiencing acute mental health difficulties. On the other hand, Teams #1, #2, and #4 were able to provide intensive contact with service users and/or their care-givers for a short duration of up to four weeks while Teams #3 and #5 were not. Three teams except Teams #1 and #5 were functioning as a 'gatekeeper' to acute inpatient services, rapidly assessing individuals with acute mental health problems and referring them to the most appropriate services. In addition, four teams except Team #1 were involved in the early discharge of inpatients. Three teams (#1, #2, & #3) were involved with compulsory admission to psychiatric hospitals. Four teams except Team #4 had the availability of crisis beds or overnight admission to inpatient services. Team #4 reported having this access at three earlier reports while reporting no access at the last two report points. These are depicted in Figure 1.

All teams generally accepted service users with diagnoses of psychosis, affective disorders, mild/moderate depressive disorders, anxiety disorders, personality disorders, social/relational difficulties, acute crises, and co-existing substance misuse disorders. All teams except Team #5 accepted service users with primary substance misuse/abuse disorders including alcohol misuse, while all four teams except Team #1 also accepted service users with diagnoses of organic disorders. Four teams except Team #2 had no exclusion criteria to screen for the acceptance of service users. Two teams (#1 and #4) received referrals from all sources such as self, family members, specialists, community service agencies, regular physician, emergency unit, police, and others. Teams #2, #3, and #5 did not accept referrals by self or family members, while Team #3 did not accept referrals from community service agencies and police in addition.

In general, the teams did not provide home visiting services over 24 hour period, 7 days a week. The individual team's working hours changed during the 18 month period, some teams expanding the working hours while others reducing the hours. Teams #3 and #4 had the same working hours the whole period ('8-15.30' on weekdays only); Team #1 expanded working hours after the first registration period from '8-15.30' on weekdays only to '8-22' on weekdays and '8-15:30' on weekends. Team #2 had working hours of '8-22' on weekdays only the first registration period and reduced after that to '8-15:30' on weekdays only for the rest of the period. Team #5 had working hours of '8-15:30' on all days for the first two registration periods and reduced to '8-15:30' on weekdays only for the rest of the period.

### Practice of the CRHT Teams

**Figure 2:**
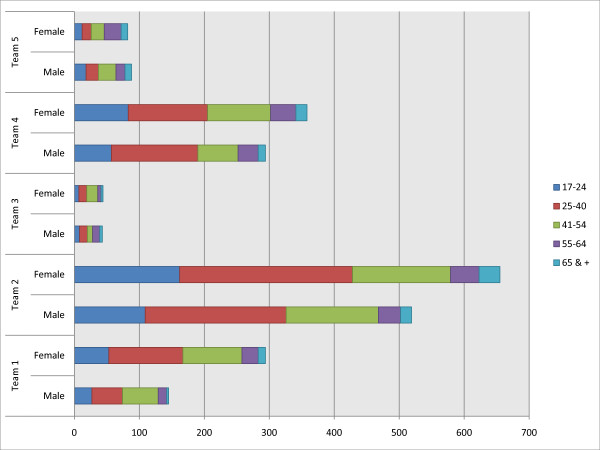
**Service user distribution in gender and age groups for the 18 months period in the CRHT teams**.

We report the services of the CRHT teams provided over the 18 months in an aggregated, summed manner based on the reports on the service questionnaires for this period. Although there were some variations from month to month revealed in our preliminary data analysis, the variations were minimal, suggesting that data in an aggregated form for the 18 months would give a comprehensive picture of the services provided by these teams. The data regarding the practice of the five teams revealed that there are in general variations among the teams in terms of the amount of services provided, referral sources, the features of services, and discharge destinations. Figure 2 shows the age and gender distribution of service users over the 18 months period, and indicates that the distributions by age for the two gender groups were not significantly different except for Team #5 in which male service users tended to be younger than the female service users at the statistically significant level. In general, the majority of service users were in the 25 to 54 age groups for both males and females.

The total numbers of services provided by the teams and the averages per month are shown in Table 5. Team #2 did not report data on referrals received as shown in this table. Teams #1, #2, and #4 had large caseloads as indicated by the total numbers of new registrations and consultations. The numbers of new patient registrations per professional staff during the 18 month period ranged from the low of 12 for Team #3 to the high of 83 for Team #2 as well as the numbers of consultations per professional staff ranging from the low of 15 for Team #3 to the high of 515 for Team #2. In addition to direct consultation services to service users, the teams also provided telephone contacts with patients or family members in varying numbers. However the telephone contacts were mostly with patients. These data suggest that for Team #2, there were at the average about 7 telephone contacts with patients in addition to 20 consultations by the Team's 9 to 14 professional staff per a given work day. In contrast, for Team #5, there were at the average less than one telephone contact and less than one consultation per a work day by the Team's 4 professional staff.

**Table 5:** Total services provided by the teams for the 18 months period & averages per month

Referrals & Services	Team 1		Team 2		Team 3		Team 4		Team 5	
	
	T^1^	A^2^	T	A	T	A	T	A	T	A
# of referrals	1104	61	0	0	172	10	2742	152	250	14
# of new registration	418	23	1163	65	81	5	488	27	71	4
# of consultation	1889	105	7207	400	107	6	2682	149	110	6
# of telephone contact with Patient	328	18	2296	128	76	4	585	33	50	3
# of telephone contact with family members	0	-	0	-	33	2	43	2	15	1

^1^T = Total for the 18 months period; ^2^A = Average per month

Patient referrals were received from various sources as shown in Table 6. The distribution in the referrals received from different sources by the teams was significantly different among the Teams (χ^2 ^= 1030.057; *df *= 24; p < .001), as was the distribution in the service users accepted by the Teams from different referral sources (χ^2 ^= 1078.385; *df *= 32; p < .001). Team #1 had more than half of its referrals by self and family members (53%), while the referrals from DPS and family physician made up 81% for Team #3, and 63% for Team #5 with two other teams (#2 and #4) with the majority of their referrals from the professionals or institutions. There may be a specific guideline, policy or culture that is at work in the communities for Team #1 that allows or encourages social networks to be the referral sources. For the four teams with both the referral and acceptance data, the overall rate of acceptance ranged from the low of 5.2% for Team #3 and 9.1% for Team #4 to the high of 37.7% for Team #1 and 52.5% for Team #5. This means that regardless of the referral sources, those who were referred to Teams # 3 and #4 had a very slim chance of being accepted as service users by these teams.

**Table 6:** Referrals to the teams during the 18 months period-Total numbers & percent within each team

Referred By		Team 1		Team 2		Team 3		Team 4		Team 5	
		
		A^1^	B^2^	A	B	A	B	A	B	A	B
DPS	N %	89 (8.0)	36 (8.5)	0 (-)	26 (2.3)	14 (12.2)	2 (33.3)	24 (3.9)	2 (3.5)	41 (18.4)	27 (23.1)
Family physician	N %	155 (13.9)	77 (18.2)	0 (-)	642 (55.7)	79 (68.7)	3 (50.0)	197 (32.1)	19 (33.9)	100 (44.8)	51 (43.6)
Municipality social services	N %	162 (14.5)	57 (13.5)	0 (-)	42 (3.6)	3 (2.6)	0 (-)	17 (2.8)	3 (5.4)	30 (13.5)	18 (15.4)
Self	N%	380 (34.0)	133 (31.4)	0 (-)	2 (0.2)	2 (1.7)	0 (-)	59 (9.6)	4 (7.1)	13 (5.8)	5 (4.3)
Relatives	N %	214 (19.2)	71 (16.8)	0 (-)	0 (-)	1 (0.9)	0 (-)	15 (2.5)	1 (1.8)	6 (2.7)	2 (1.7)
Emergency ward	N%	65 (5.8)	28 (6.6)	0 (-)	220 (19.1)	11 (9.6)	1 (16.7)	73 (11.9)	6 (10.7)	3 (1.4)	2 (1.7)
Police	N %	0 (-)	0 (-)	0 (-)	4 (0.4)	0 (-)	0 (-)	0 (-)	0 (-)	0 (-)	0 (-)
Psychiatric hospital	N%	47 (4.2)	20 (4.7)	0 (-)	33 (2.9)	2 (1.7)	0 (-)	27 (4.4)	1 (1.8)	18 (8.1)	7 (6.0)
Physician in charge at psychiatric hospital	N %	3 (0.3)	1 (0.2)	0 (-)	148 (12.8)	0 (-)	0 (-)	86 (14.0)	5 (8.9)	8 (3.6)	4 (3.4)
Others	N %	2 (0.2)	0 (-)	0 (-)	36 (3.1)	3 (2.6)	0 (-)	115 (18.8)	15 (26.8)	4 (1.8)	1 (0.9)
TOTAL	N	1117	423	0	1153	115	6	613	56	223	117

^1^A = Number of Referrals Received; ^2^B = Number of Referrals Admitted as Patients

**Figure 3:**
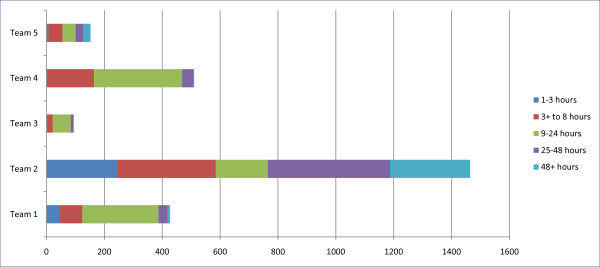
**Distribution in the categories for the time between referral to initial contact during the 18 months for the CRHT teams**.

Figure 3 shows the distribution in the average time between the receipt of referrals and initial contact with potential service users. There was a significant difference among the five teams in this distribution (χ^2 ^= 872.951; *df *= 16; p < .001). The majority of referrals were contacted within 24 hours in Team #1 (91%), Team #3 (89%), and Team #4 (91%), while Teams #2 had the largest proportion being contacted after 24 hours (47%) followed by Team #5 that had 34% in the categories of 25 hours or longer.

**Figure 4:**
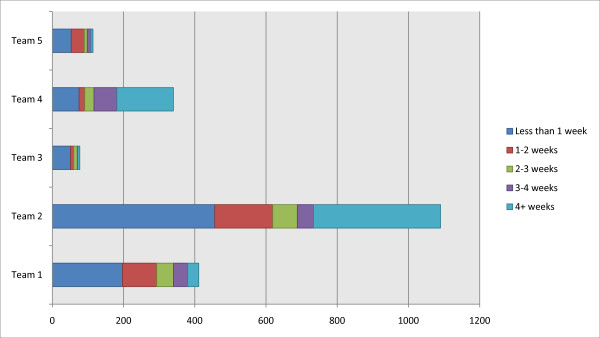
**Distribution in the categories for the duration of service contact during the 18 months period for the CRHT teams**.

The distributions in the length of the duration of service contact are shown in Figure 4, and are statistically different among the Teams (χ^2 ^= 347.911; *df *= 16; p < .001). Team #4 had the largest proportion in the 'longer than 4 weeks' category (47%) followed by Team #2 with 33%, while Team #3 had the largest proportion in the 'less than 1 week' category (66%) among the teams. All teams except Team #4 had more than 50% of their service users with less than 2 weeks of service duration.

The discharge destinations during the 18 months period by the CRHT teams are shown in Table 7. The distributions are significantly different among the teams (χ2 = 364.497; *df *= 24; p < .001-The χ2 calculation for this table excluded the 'None' category as this category had only 5 cases across the five teams.). About half of the discharge destinations for the teams were to general practitioners (the patients' regular physicians) with some variations among the teams in the discharge destinations of the remaining half. Team #3 discharged 42% of their service users to DPS (inpatients or day care), while Team #5 discharged 35% of its service users to community health centers.

**Table 7:** Discharge destinations during the 18 months Period

Discharge destination	Team 1 N (%)	Team 2 N (%)	Team 3 N (%)	Team 4 N (%)	Team 5 N (%)
Psychiatric hospital	54 (10.0)	81 (7.4)	3 (3.1)	1 (0.3)	10 (8.9)
DPS-inpatient unit/crisis-unit	23 (4.1)	115 (10.4)	30 (31.3)	26 (7.7)	3 (2.7)
DPS- daycare or outpatient clinic	106 (19.0)	114 (10.3)	10 (10.4)	13 (3.9)	6 (5.3)
General Practicioner	261 (46.7)	673 (61.1)	46 (47.9)	246 (73.2)	52 (46.0)
Community health centre	89 (15.9)	70 (6.4)	3 (3.1)	15 (4.5)	40 (35.4)
Private Practice Psyciatrist/Psychologist	8 (1.4)	8 (0.7)	0 (-)	20 (6.0)	0 (-)
Others	17 (3.0)	40 (3.6)	4 (4.2)	13 (3.9)	1 (0.9)
None	1 (0.2)	1 (0.1)	0 (-)	2 (0.6)	1 (0.9)
TOTAL	559	1,102	96	336	113

## Discussion

The Reform of mental health-care in Norway called for a major increase in the funding of mental health-related services, as well as a major re-organization of these services. The Reform has been an important initiative and has invested in fundamental changes in the way mental health services are provided [5]. However, implementation of new service models is often met with barriers that are fundamentally rooted in the dominant culture of mental health-care including the resistance to shifting from the biomedical paradigm orientation to humanistic, person-oriented, and social-oriented mental health services and from professional-control to service-user orientation [11]. Furthermore, although the Reform has placed a major emphasis on the establishment of CRHT teams, a great variety of specialized community mental health teams are being established in many localities causing complex challenges for overall service organization, leadership and management [2,3,9]. At present, it is difficult to determine the dynamics between CRHT teams and other forms of specialized mental health teams.

This study offers an evidence of the profiles and practices of five CRHT teams in a health region in Norway by identifying basic characteristics of the teams as well as the services provided. CRHT teams have been established rapidly throughout Norway since the national strategy was initiated in 2004 [9,12]. Although the establishment and operation of CRHT teams were recommended to follow a set of practice guidelines established at the national level [6,11], this study reveals a multifaceted and varied picture of the crisis resolution and home treatment teams in community health services. However certain common features can be elicited from the results. They were all an integral part of the larger mental health organisations and based in the local community mental health centres (DPS). They all had response time between 24-48 hours, meaning that all patient referrals were assessed within two days. Although this standard regarding the response time frame is much longer than the standard set in the UK, it is within the standard set in Norway for CRHT teams, suggesting different approaches to crisis-care at the national levels. However, the data showed that at least one third of the cases were responded within the 4 hour frame used by the UK. They also had a common practice of a named coordinator for each patient and reported to be able to provide an alternative to in-patient treatment in hospitals for people experiencing acute mental health problems. Another common feature was that none of the teams offered a 24-7 gate keeping service. In general, no exclusion criteria were applied for accepting patients, and persons with various mental health problems were serviced. These common features in the CRHT teams suggest that minimally CRHT teams operate with structures to address mental health crises from the community mental health perspective. However, from the policy perspective regarding the gate-keeping role of CRHT teams, the currently prevailing operating hours of the teams greatly limit their effectiveness for gate-keeping. One major issue common to the services of the CRHT teams is the under-representation of the older adults as service users. The older adults were in general excluded from this service, suggesting a continuing problem in providing community mental health services to this group of population. This problem is emphasised in the final evaluation of the National Action Plan for Mental Health [13] revealing that the majority of white papers and political strategies focus on the adult population below retirement age and do not take adequate account of the needs of older people experiencing mental health problems. The community mental health centres' patient lists represented only 7% of patients being over 60 years old along with those over 70 years more or less being absent.

As reported in international studies [3,4,7] there are considerable variations in both the team profiles and services. The teams seem to address diverse service demands, work under dissimilar service policies and procedures, and/or have different service mandates and priorities. While the teams were multidisciplinary, they work under a different set of caseload demands and the modes of their professional practice seem variant. In addition, there were also divergence in team structures, such as staffing, working hours, post graduate training, team organisation, and travel time. For example, a service user could expect to receive help for just a few days in one team while in other teams he or she could be offered support from the professionals for several months, although the majority of service duration remains to be two weeks which is the standard recommended for CRHT teams. The variations in the teams' services in the data on the percent of referrals accepted by the teams, the discharge destinations, the rates of acute hospitalization referrals, and the referral sources also suggest that CRHT teams may operate with the team-specific guidelines regarding these issues, resulting in a service user to attain different outcomes depending upon his/her residence location. Such variations may obviously be related to the diversity in the local mental health enterprises' traditions and cultures, team make-up, population served, and team mandates. However, three elements may transcend the local and situational contingencies in influencing such variations. These are perspectives on the nature of mental health crisis, the varied policies in mental health care, and the nature of home-based mental health services.

First, since the clinical focus of CRHT teams is mental health crisis, there is a need to have a common understanding and definition of mental health crisis in the context of mental health services and community health care. As described in the literature [3,14-16], mental health crisis defined by biomedical understanding of crisis is insufficient for these teams as it needs to be viewed as human experiences embedded within the situations and contexts of experiences. This means that mental health crisis has to be viewed from the psychological, social, and phenomenological perspectives that go beyond the medical, pathological perspective. This also means that the processes and interventions used by CRHT teams may involve various psychological, communicative, and social strategies going beyond the standard protocols of mental health services, making their practice complex. As CRHT teams get established, teams may follow different paths to establish common understandings and processes for dealing with mental health crises in the community, resulting in a great variation. Therefore, the variations reported in this study point to the need to examine how CRHT teams establish their approaches to mental health crises and fashion their services.

Secondly, the field of mental health services is entrenched with many contradictory policies that govern the way services are provided. For example, on one hand clinicians are expected to be user-oriented and follow individual's preferences, while on the other they are required to adhere to national guidelines of best practice and comprehensive documentation procedures that have great impact on encounters with individual service users and their families, and which may jeopardize the principle of user-orientation. Another example is the debate in the clinical community as well as in the political arena regarding the essence of good acute mental health service. This debate in Norway often focuses on the concern regarding clinical responsibilities of mental health professionals, especially in relation to the role of mental health nurses in providing crisis mental health care including assessment and treatment vis-à-vis those of psychiatrists or clinical psychologists. Such contradictions and controversies often result in the resolution of practice issues on an ad hoc basis or through the dynamics of local situations, which can produce variations in practice from one team to another and from one situation to others. This variation in teams and local differences in team provision is also reported from UK in the National Audit Office Report [1].

Thirdly, the home-based mental health care is influenced by the dynamics of contextual aspects of home not only as the situ of crises but also as the place in which effective assessment and treatment must occur. Working in people's homes can be challenging to health professionals. It involves being in a venture of unpredictable relations and unpredictable scenarios, and requires of the professionals to draw on different professional skills and competencies from those being used in professional-controlled settings that have readily available professional support for practice [15]. The support and supervision available for practice development in CRHT teams may vary and may have impact on the procedures and practices developed by individual teams.

## Conclusions

The results of this study show that in spite of national guidelines and recommendations for the establishment and practice of CRHT teams the team profiles and standards of practice vary a great deal among the 5 participating teams. A general standardization regarding response time, opening hours, key areas of functioning, and types of mental health problems handled has been found. However the practices are by no means on the level of the UK standards and may well be a result of the lack of formal guidelines and specific CRHT training in Norway or the differing policy decisions regarding the standards. There is a need to consider the use of the term CRHT for cross-national comparisons as well as for country-specific understandings, as revealed in this study the operationalization of CRHT teams in local settings is quite varied.

This research reveals variations in referrals, duration of services, and general team policies identify the need for further research on the characteristics of home treatment, the backgrounds for the variety in interventions used by CRHT teams as well as exploring service users' and family members' preferences and perspectives and whether their expectations are taken much into account. There also is a need to examine the actual practices and processes used by CRHT teams' professional staff as well as the organizational structures that impinge on the operation of such teams. While it can be argued that the results are very specific to the research site, the study raises many questions regarding the ways CRHT teams are established structurally and how CRHT teams carry out their services to target populations. Studies of local experiences such as this study can be a rich foundation for further research regarding both the structural and process elements that impact on the quality of services of CRHT teams. The data can be used as the initial baseline for benchmarking for CRHT teams in Norway. The results of this longitudinal study suggest that there is a need to view benchmarking for CRHT teams according to urban-rural differences. The next step needs to be an in-depth examination of the quality and cost of services in CRHT teams against the structural and process variations.

One of the critical shortcomings of this study is the aggregate nature of the data. The reliability of the reported data for the surveys in the aggregate forms can be questioned as it depends completely on the reporters' accountability, even though we tried to assure such accountability through the regular, periodic conferences with the reporters throughout the period of data collection. A more carefully designed study using individual service user data is thus recommended. Further refinement of the survey tools is recommended so that the team profile questionnaire would strictly focus on the structural issues while the service questionnaire focuses on the on-going practices and services by the team. There is a need to consider including other structural issues within the team profile questionnaire such as the workings of teams, the nature of supervision and leadership within teams, and the nature of policies established within teams regarding various aspects of services as the current tool only addressed team meeting and the mandates for response time. Such revision will be critical in applying these questionnaires to establish benchmarking for CRHT teams in future studies. Given the degree of stability found in our study with the team profile data, it is recommended that an annual data collection for the team profile with a monthly data collection for the service data for a period of 12 months would seem appropriate. These survey instruments developed with an input from a UK researcher with a careful refinement would be appropriate for cross-national applications with some considerations to address local characteristics.

## Competing interests

The authors declare that they have no competing interests.

## Authors' contributions

BK, MB, and HSK contributed to the concept and design of the research. ME collected and managed the data, contacted the research participants for data completion, and worked on the initial draft. BK, MB, and HSK were involved in writing, reviewing, revising, and completing the manuscript. All authors have read and approved the final version. All authors have participated sufficiently in the work to take public responsibility for appropriate portions of the content.
